# Supplementary material for: Silicon single-photon avalanche diodes with nano-structured light trapping
Source: Nat Commun. 2017 Sep 20;8:628. doi: 10.1038/s41467-017-00733-y (PMC5607005; doi:10.1038/s41467-017-00733-y)
Supplement: Supplementary file 2 — Supplementary Information [file 41467_2017_733_MOESM2_ESM.pdf]

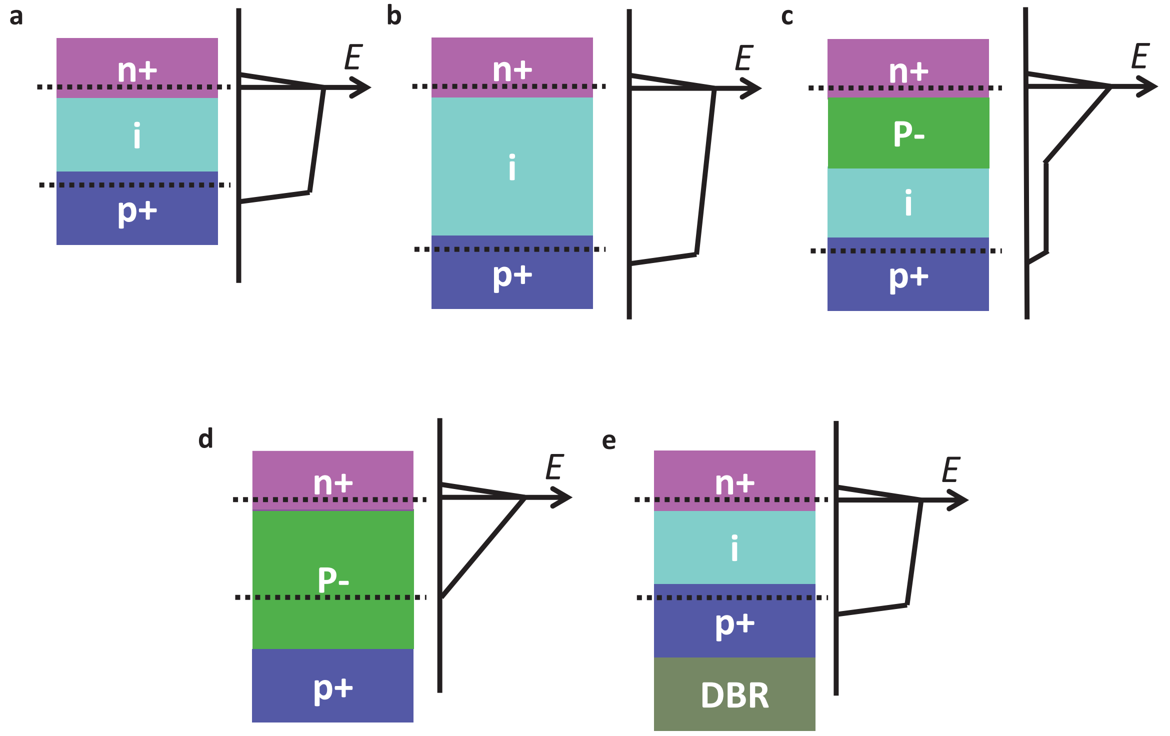

Supplementary Figure 1 | Schematics and electric field distributions for respective SPAD designs. Dashed lines denote depletion region boundaries. **a**, Control device thin-junction SPAD based on PN junction. **b**, Absorption enhancement based on thicker avalanche region. **c**, Absorption enhancement based on extended depletion region to drift carriers to the avalanche region. **d**, Absorption enhancement based on extended diffusion region. **e**, Resonant-cavity-enhanced structure.

(a) (b)

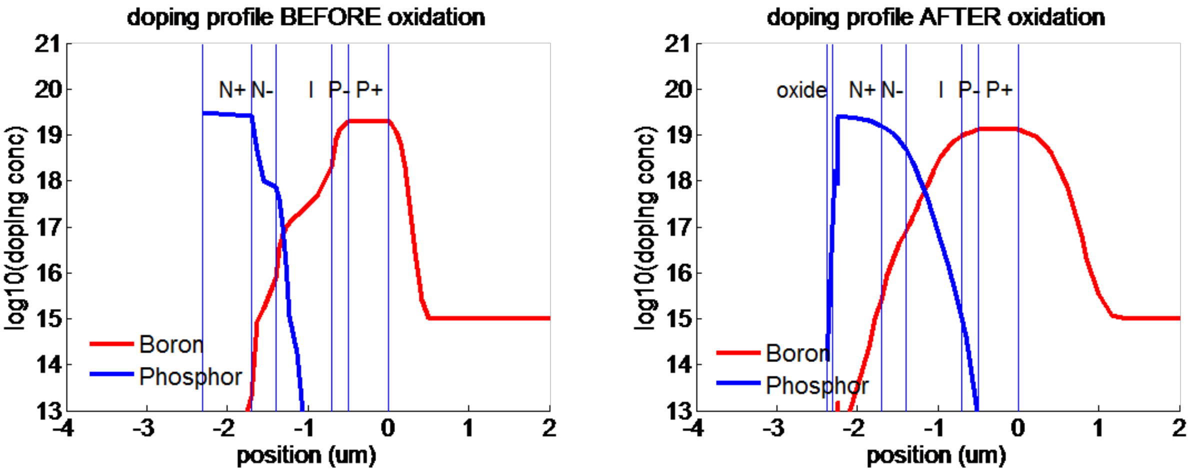

Supplementary Figure 2 | Simulated epitaxial doping profile before (a) and after (b) thermal oxidation. Due to dopant diffusion, after the thermal oxidation at 1000 °C, there is a higher doping in intrinsic region. So the depletion width decreases, which reduces the SPAD breakdown voltage.

Supplementary Note 1 | Nano-structure reflection measurement

Inverse pyramid nano-texturing could be treated as a two dimensional grating, so in reflection measurement, it is expected to have a resonance valley in the spectrum. As in Supplementary Figure 3(a), with photon injection angle shifts, the resonance valley has a clear shift. Across the spectrum, reflection is below $10\,\%$ when θ is between $0°$ and $30°$. This shows that surface texturing serves as an anti-reflection layer. In comparison between control and light-trapping SPAD as in Supplementary Figure 3(b), the control SPAD has higher reflection as well as sharp resonances, suggesting a strong vertical resonance between SOI substrate and top surface. Light-trapping SPAD, however, has lower reflection and much weaker resonance, suggesting that photons are coupled to waveguiding mode and are horizontally propagating instead of creating a vertical resonance.

(a) (b)

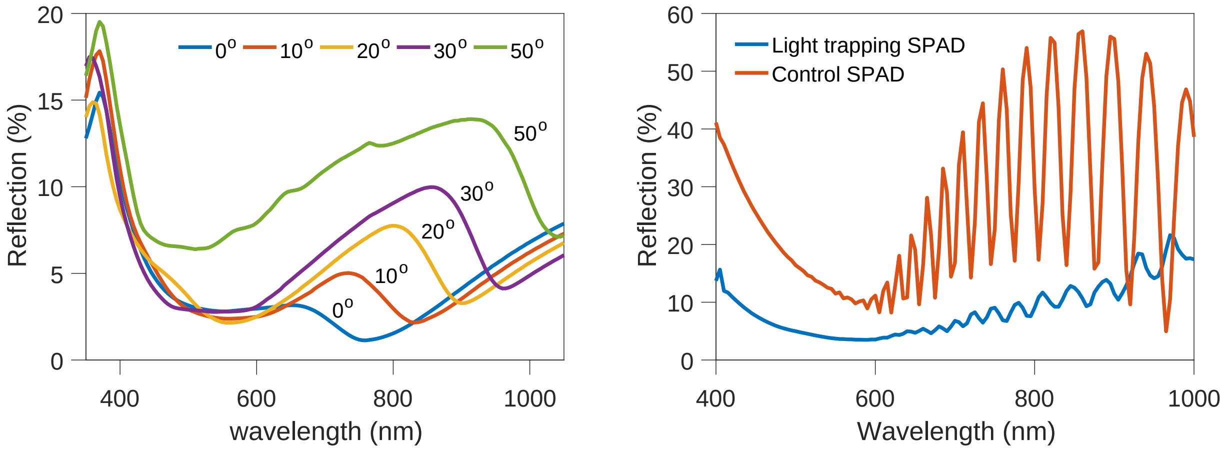

Supplementary Figure 3 | Nano-structure reflection measurement. **(a)**, Reflection measurement of $700\,\mathrm{nm}$ period inverse pyramid nano-structure on Si substrate at different θ. **(b)**, Reflection measurement of light-trapping SPAD ($850\,\mathrm{nm}$ period inverse pyramid nano-structure on SOI substrate) and control device (no surface texturing with SOI substrate) at vertical incidence.

Supplementary Note 2 | External quantum efficiency angle dependent measurement

The light trapping SPAD angular dependence on external quantum efficiency (EQE) has been measured in a rotational stage with the ambient light blocked. ϕ of $0°$ and $45°$ are chosen as directions for incoming photons. The results have been normalized to EQE of light trapping SPAD at vertical injection angles. As shown in Supplementary Figure 4, at $850\,\mathrm{nm}$ wavelength, photons will have a fairly decent absorption at different angles of incidence. This is because the nano-structure has been optimized for this wavelength. At different incoming angles, the absorption of $980\,\mathrm{nm}$ wavelength remains above $50\,\%$ of normal EQE and we could further optimize it by changing the nano-structure period and pattern (e.g. from square lattice to hexagonal or more complicated lattice structure).

(b)

(a)

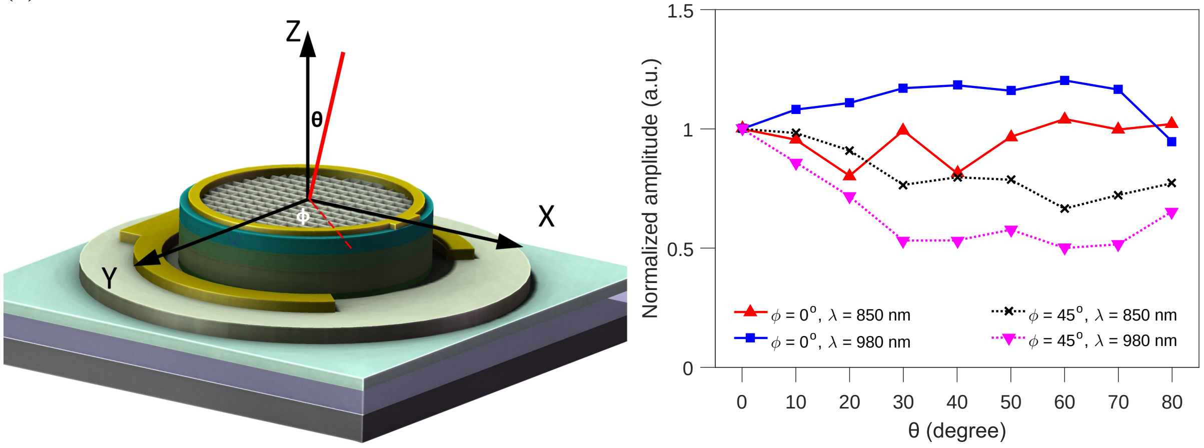

Supplementary Figure 4 | External quantum efficiency angle dependent measurement. **(a)**, Schematic representation of external quantum efficiency angle-dependent measurement. **(b)**, Measured results for two wavelengths in two directions.

Supplementary Note 3 | Estimation of the uncertainty of the Photon Detection Efficiency

We derived the uncertainties of each measurement steps to calculate the final uncertainty of the PDE. The PDE of a SPAD can be calculated by the formula, $-\frac{1}{\mu}\ln\frac{1-P_1}{1-P_0}$, where P_1 denotes the probability of detecting an avalanche signal per gate with pulsed illumination; P_0 denotes the probability of detecting an avalanche signal per gate without illumination; and μ denotes the average photon number of each laser pulse. Assuming that all of the uncertainties of the components were independent, the uncertainty of PDE is expressed as:

$$u_{PDE} = \sqrt{\left(\frac{\partial PDE}{\partial p_1}\right)^2 (u_{P_1})^2 + \left(\frac{\partial PDE}{\partial p_0}\right)^2 (u_{P_0})^2 + \left(\frac{\partial PDE}{\partial \mu}\right)^2 (u_\mu)^2} \quad (1)$$

where u denotes the standard deviation of each single variable. Equation (1) means the standard deviation of a function is the geometrical mean of the standard deviations of all variables weighted by the partial derivatives.

Uncertainty of the Photon Count/Dark Count Probability In a single measurement, we use an ultra wide band digital oscilloscope to randomly sample the output signal of the SPAD. At each gate sampled, we check whether an avalanche pulse is detected in that gate. When a total of C_t gates are checked and in which C_{p_1} avalanche pulses are found under illumination or C_{p_0} avalanche pulses are found without illumination, then P_1 and P_0 can be calculated by $P_1 = C_{p1}/C_t$ and $P_0 = C_{p0}/C_t$ respectively. The C_t is constant in our measurements. The C_{p_1} obeys a binomial distribution, therefore the uncertainty of C_{p_1} is $\sqrt{C_t P_1(1-P_1)}$. So the uncertainty of P_1 will be:

$$u_{P_1} = \frac{\sqrt{C_t P_1(1-P_1)}}{C_t} = \sqrt{\frac{C_{p_1}(C_t - C_{p_1})}{C_t^3}} \quad (2)$$

In the same way, the uncertainty of P_0 will be:

$$u_{P_0} = \frac{\sqrt{C_t P_0 (1 - P_0)}}{C_t} = \sqrt{\frac{C_{p_0}(C_t - C_{p_0})}{C_t^3}} \tag{3}$$

Uncertainty of the Average Photon Number The average photon number per pulse is calculated with the following equation, $\mu = kP$, where P denotes the total laser beam power measured by optical power meter and k is a ratio determined by the laser pulse repeat rate, the photon energy, the beam spot size at 13.5% maximum intensity, the active area of our SPADs, and the alignment of SPAD and Gaussian beam center. The laser beam power P is measured by an optical power meter from Thorlabs Inc., PM200, with sensor head S130VC. The datasheets indicate that the measurement uncertainty of S130VC is $3\,\%$ and the measurement uncertainty of PM200 is $0.5\,\%$ of full scale ($97\,\mathrm{nW}$ in our experiments). The beam spot size at 13.5% maximum intensity is measured to calculate the intensity at beam center, where our SPAD is placed. Thus the power portion that actually illuminates the SPAD can be calculated. The uncertainty of beam spot size contributes little to the uncertainty of average photon number per pulse. First, the beam spot size at 13.5% maximum intensity is about $3\,\mathrm{mm}$ in our experiment, which is significantly large compared to the $20\,\mathrm{\mu m}$ diameter of the SPAD. Second, the beam spot size is measured using a beam profiler (BC-106N), and if we fit the spot image in Matlab we will see the relative uncertainty will be about only $0.2\,\%$. The accuracy of active area of our SPADs is guaranteed by design and fabrication. The wavelength of the laser, which is measured by an optical spectrometer, also has high accuracy. The laser pulse repetition rate is quite stable and accurate. The significantly large beam spot size reduces the requirement of aligning the SPAD and beam center. So the alignment also contributes little to the uncertainty of average photon number per pulse. Also the not so perfect alignment

will merely cause under estimation of photon detection efficiency. In summary, the uncertainty of laser beam power is the main part of uncertainty of average photon number per pulse, and k can be treated as constant. Thus the uncertainty of average photon number per pulse is :

$$u_\mu = k u_P = \frac{\mu u_P}{P} = \mu \sqrt{\left(\frac{0.005 * 97\,\mathrm{nW}}{P}\right)^2 + 0.03^2} \tag{4}$$

We can get the uncertainty of Photon Detection Efficiency using equations (1), (2), (3) and (4).

Supplementary Note 4 | Estimation of the Uncertainty of the Full Width Half Maximum of Timing Jitter

The full width half maximum (FWHM) of timing jitter is read out from the histogram of photon arriving time, which is the time from the laser pulse to the time the avalanche signal crosses the threshold. Let C_{max} be the maximal counts of the histogram. Draw a $0.5C_{max}$ horizontal line, which will cross the distribution on both left and right of the peak. The distance between the two cross points can be read out as FWHM. But the time bin is $5\,\mathrm{ps}$, and the statistical fluctuation of counts on each bin is quite large, so some fitting is needed to get a more accurate read out of FWHM. It's hard to find a function to describe the whole distribution of photon arriving time detected by SPAD, so global fitting is not feasible. Instead, we choose a local fitting method. Several data points near the two cross points are chose manually and fitted by a linear function $t = a + bc$ separately, in which c is counts of photon arriving at t, and a, b are the coefficients to be fitted. The fitting result contains a_1, b_1 with uncertainty u_{a_1}, u_{b_1} for the left cross point and a_2, b_2 with uncertainty u_{a_2}, u_{b_2} for the right cross point. New cross points are calculated based on the fitting result, and a more accurate FWHM can be calculated. Let $c_1 = c_2 = 0.5C_{max}$, and uncertainty $u_{c_1} = u_{c_2} = \sqrt{0.5C_{max}}$, the uncertainty of FWHM will be:

$$u_{FWHM} = \sqrt{\left(\frac{\partial t}{\partial a_1}u_{a_1}\right)^2 + \left(\frac{\partial t}{\partial b_1}u_{b_1}\right)^2 + \left(\frac{\partial t}{\partial c_1}u_{c_1}\right)^2 + \left(\frac{\partial t}{\partial a_2}u_{a_2}\right)^2 + \left(\frac{\partial t}{\partial b_2}u_{b_2}\right)^2 + \left(\frac{\partial t}{\partial c_2}u_{c_2}\right)^2} \tag{5}$$

$$= \sqrt{(u_{a_1})^2 + (0.5C_{max}u_{b_1})^2 + (b_1 u_{c_1})^2 + (u_{a_2})^2 + (0.5C_{max}u_{b_2})^2 + (b_2 u_{c_2})^2}$$

The uncertainty of FWTM and tail can be calculated in the same way.
